# Staff SARS-CoV-2 Seroprevalence and Mental Health as Key Factors in University Response to COVID-19 Pandemic

**DOI:** 10.3389/fpubh.2021.689919

**Published:** 2021-06-16

**Authors:** David G. Lopes, Ana Rita Henriques, Margarida Santos-Dias, Catarina Nunes-da-Silva, Juliana Gonçalves, Rute D. de Sousa, Saba Abdulghani, Jair Eletério, Sofia Jacinto Braga, Helena Soares, Jaime C. Branco, Helena Canhão, Ana M. Rodrigues

**Affiliations:** ^1^Comprehensive Health Research Centre, NOVA Medical School, Universidade NOVA de Lisboa, Lisbon, Portugal; ^2^EpiDoC Unit, Chronic Diseases Research Center (CEDOC), NOVA Medical School, Universidade NOVA de Lisboa, Lisbon, Portugal; ^3^Comprehensive Health Research Centre, Lisbon Institute of Global Mental Health, Lisbon, Portugal; ^4^Human Immunobiology and Pathogenesis Laboratory, Chronic Diseases Research Center (CEDOC), NOVA Medical School, Universidade NOVA de Lisboa, Lisbon, Portugal; ^5^Serviço De Reumatologia Do Hospital Egas Moniz—Centro Hospitalar Lisboa Ocidental (CHLO-E.P.E.), Lisbon, Portugal; ^6^National School of Public Health, Universidade NOVA de Lisboa (UNL), Lisbon, Portugal

**Keywords:** coronavirus, SARS-CoV-2, serology testing, adaptation to COVID-19, public health, academic environment, mental health

## Abstract

**Background:** In response to rapid global spread of the newly emerged coronavirus disease 2019 (COVID-19), universities transitioned to online learning and telework to decrease risks of inter-person contact. To help administrators respond to the COVID-19 pandemic and better understand its impacts, we surveyed SARS-CoV-2 seroprevalence among NOVA University employees and assessed community mental health.

**Methods:** Data were collected from voluntary participants at six NOVA University locations, in the Lisbon metropolitan area, from June 15–30, 2020. All subjects provided written informed consent. Of 1,627 recruited participants (mean age 42.0 ± 12.3 years), 1,624 were tested. Prior to blood collection, participants completed a questionnaire that assessed: COVID-19 symptoms during the previous 14 days, chronic non-communicable diseases, chronic medication, anxiety, and depression symptoms. SARS-CoV-2 serology tests were then performed, and results communicated approximately 4 days after blood draw. Participants with positive serology tests were contacted to assess COVID-19 symptoms since February.

**Results:** Estimated prevalence of SARS-CoV-2 IgG antibodies was 3.1% (*n* = 50), of which 43.5% reported symptoms in the previous 4 months. The Medical School had the highest seroprevalence (6.2%). Participants reported having at least one chronic disease (63.7%), depression-like symptoms (2.1%), and anxiety symptoms (8.1%). Rates of depression and anxiety symptoms were significantly higher in women, with sleep hours and occasional alcohol consumption negatively associated with depression. Male gender, older age, and sleep hours negatively associated with anxiety symptoms. School of employment and presence of comorbidities positively associated with anxiety.

**Conclusion:** By measuring seroprevalence of SARS-CoV-2 antibodies among NOVA employees and assessing subjects' mental health, we aim to help administrators at European public universities in urban areas, such as Lisbon, Portugal, better understand the needs of their communities. This study resulted in implementation of a stricter contingency plan in the Medical School, while other schools continued to follow Government mitigation guidelines. These findings may also guide the development of tailored strategies to ensure physical and mental health of the academic community during this pandemic crisis. We conclude that, together with COVID-19 contingency plans, psychological support services and facilities to help people effectively face pandemic-associated challenges and minimise anxiety and depression should be implemented.

## Introduction

In December of 2019, a new human disease caused by the severe acute respiratory syndrome coronavirus-2 (SARS-CoV-2) was identified in Wuhan, China (1). Since then, this disease, known as coronavirus disease 2019 (COVID-19), has rapidly spread, causing a worldwide pandemic, with over 115 million infections and 2.5 million deaths. Portugal has reported over 800,000 infections and 16,000 deaths (2). In an attempt to control rapid spread of the virus, governments around the world took unprecedented steps—issuing shelter-in-place orders and closing all non-essential businesses, including institutions of higher education (3). For example, at the start of this pandemic, beginning in early March 2020, Portugal imposed a national lockdown, with people only resuming in-person work at the end of May 2020. Accordingly, the COVID-19 outbreak and its rapid global spread affected society on multiple levels, including its educational systems. In particular, the rapid emergence and spread of this disease necessitated a quick transition to online learning and telework to minimise the risk of inter-personal contact and subsequent COVID-19 infection (4, 5).

Due to the variable range of clinical presentations associated with COVID-19, in some cases including absence of any infection-related symptoms, disease incidence at higher education institutions is difficult to assess. Although great focus has been placed on understanding and minimising the risks of COVID-19 for students (6–8), only limited research efforts have focused on understanding and addressing the needs of university employees. The COVID-19 pandemic has put significant stress on these individuals, many of whom are worried about the risk of being infected in the workplace and feeling both increased financial pressure and insecurity about the future (6, 9). Additionally, efforts to follow World Health Organization directives to ensure health and safety of the academic community have created significant organisational demands (10, 11) that likely imposed an increased burden on university employees. Researchers and professors may also feel heightened concerns regarding the quality of their research and educational services, as abrupt changes were made without sufficient preparation. The unprecedented home lockdown experience also led to several reports of increased anxiety and depression symptoms (12, 13) and a subsequent increase in psychiatric medication (14). Although, the impact of lockdown on mental health of university employees is largely unknown, understanding this will be critical for facing the myriad academic challenges generated by the COVID-19 pandemic.

To assess the direct risk of SARS-CoV-2 infection faced by university employees, we aimed to measure exposure to this virus among NOVA faculty and staff. Moreover, to better understand indirect pandemic-associated risks to employee health, we sought to measure the mental health of professors, researchers, and staff members during the process of adapting to COVID-19 measures and preparing for the following school year. To achieve these goals, we surveyed NOVA employees and assessed: (1) prevalence of antibodies against SARS-CoV-2; (2) general health, including the prevalence of diagnosed chronic diseases, depression, and anxiety symptoms; and (3) specific factors associated with these symptoms. Through this study, we aim to better understand the comprehensive health risks faced by faculty and staff at a university in a European capital highly affected by COVID-19.

## Materials and Methods

### Setting

NOVA University includes nine independent schools, plus the Rectory and Social Services, located in different sites across the greater Lisbon metropolitan area. The individual sites are as follows: Sciences and Technology School (FCT), Social and Human Sciences (FCSH), School of Business and Economics (SBE), Medical School (NMS), School of Law (FD), Information Management School (IMS), Hygiene and Tropical Medicine Institute (IHMT), National Public Health School (ENSP), Chemical and Biological Technology Institute (ITQB), the Rectory (RUNL), and Social Services (SAS). As of December 31, 2019, NOVA employed a total of 3,308 workers, including 2,363 Professors/Researchers and 945 Non-Professors/Non-Researchers (Table 1) (15).

**Table 1:** Characterisation of NOVA University workers.

	**Study population 2019 (*n* = 3,308)**	**Study sample 2020 (*n* = 1,624)**
**Sex**		
Female	1,829 (55%)	1,072 (66.0%)
Male	1,479 (45%)	552 (34.0%)
**Age Group**		
<30	241 (8%)	296 (18.3%)
(30, 39)	719 (22%)	438 (27.1%)
(40, 49)	997 (30%)	413 (25.6%)
(50, 59)	841 (25%)	309 (19.1%)
≥60	510 (15%)	160 (9.9%)
**Faculty/Institute**		
National Public Health School	67 (2%)	40 (2.6%)
Medical School	801 (24%)	240 (15.0%)
Social and Human Sciences School	620 (19%)	197 (12.3%)
Sciences and Technology School	753 (23%)	447 (28.0%)
Law School	69 (2%)	31 (1.9%)
Hygiene and Tropical Medicine Institute	109 (3%)	46 (2.9%)
Chemical and Biological Technology Institute	195 (6%)	289 (18.1%)^#^
Rectorate	172 (5%)	113 (7.1%)
Business and Economics School	438 (13%)	195 (12.2%)
Information and Management School	84 (3%)	–
**Occupational group**		
Professors/Researchers	2,363 (70%)	1,272 (81.6%)
Non-Professors/Non-Researchers	945 (30%)	287 (18.4%)

# *Chemical and Biological Technology Institute (n = 225) + Experimental and technological Biology Institute (IBET) (n = 67)*.

*Sample size is not constant due to missing values in: Age group (n = 1,616), Faculty/Institute (n = 1,598), and Occupational group (n = 1,559)*.

### Recruitment

**Figure 1:**
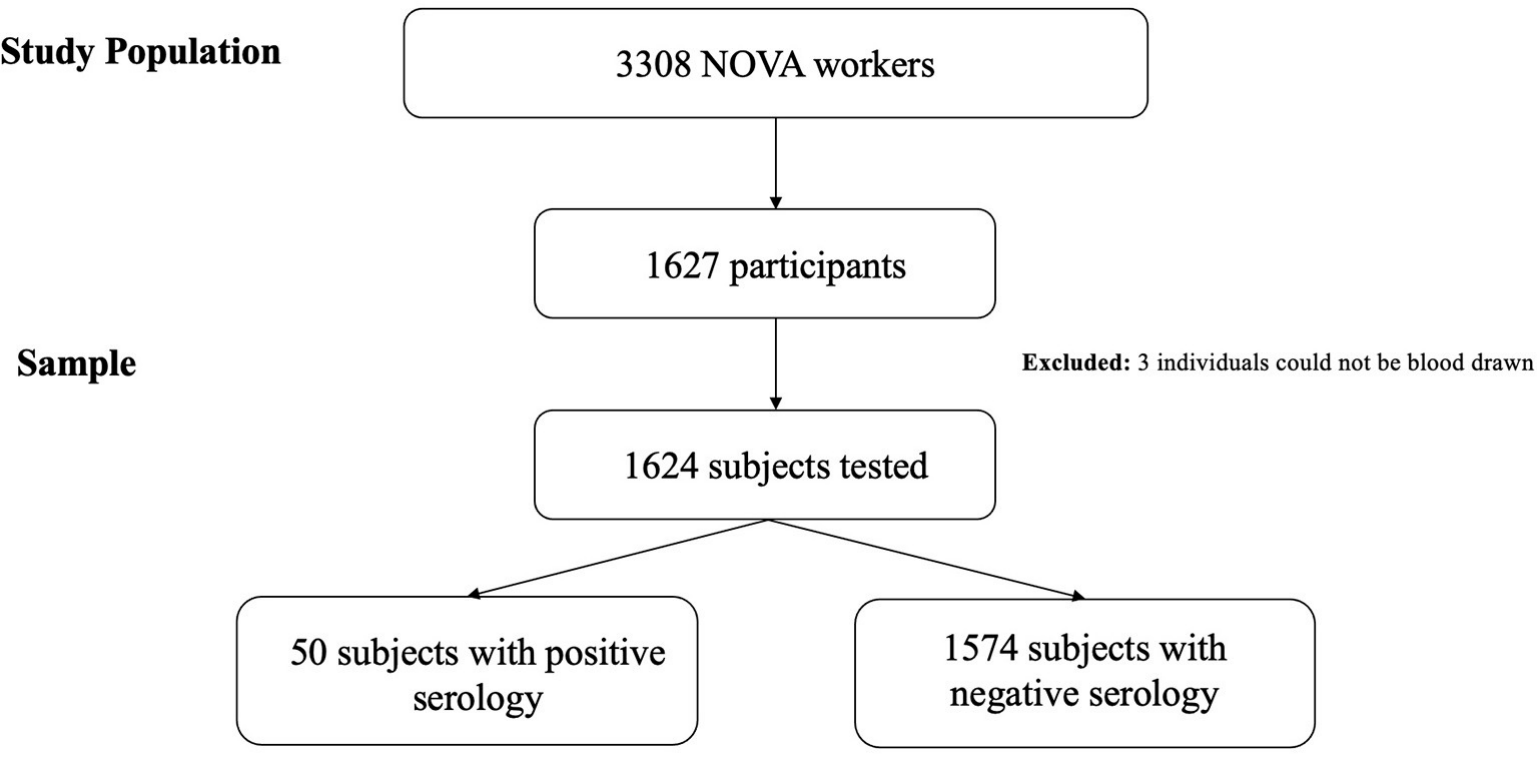
Flowchart of study design to measure SARS-CoV-2 seroprevalence and mental health among NOVA University employees.

In this observational cross-sectional study, the target population consisted of employees—professors, researchers, and administrative and operational staff—of the nine schools within NOVA University, plus those in the RUNL and SAS. The serosurvey was performed from June 15–30, 2020, by a multidisciplinary team composed of doctors, nurses, and research assistants at six locations: the RUNL, NMS, FCT, ITQB, IBET, IHMT, and SBE. Recruitment began with an online invitation from each school's communications department (*n* = 3,308), wherein an email for registry was made available. Registration was voluntary and only required the candidate's name, email, and/or phone number. Only the research team had access to participants registration to ensure that NOVA worker privacy was protected in their organisational environment. Along with the invitation email and registration form, we sent two written informed consent forms: one for the serological test and collection of epidemiological and clinical information and another for blood sample storage at NOVA's Biobank and use in future research. Included in the email was also a link to the epidemiological and clinical questionnaire. For underaged participants, written informed consent to participate in this study was provided by the participant's legal guardian/next of kin. Participants were given the opportunity to select the location (one of the six available surveying sites) and time (between June 15–30) of the serological testing and epidemiological study. A total of 1,627 workers participated in the study, and 1,624 were tested (Figure 1). For information regarding the number of days spent on each location and the number of participants tested per day, please see Supplementary Material section file.

### Procedure

At the serological testing site, participants had their temperature taken and were asked if in the previous 14 days, they had experienced any of the following symptoms: cough, fever, lack of smell or taste. If one of these was reported, the subject was invited to undergo a diagnostic reverse transcription (RT)-PCR test, free of charge. Research assistants then collected the epidemiological questionnaire and informed consent forms, addressed any questions related to the study and/or COVID-19, and directed participants to the blood collection stations. Subjects who did not bring a completed epidemiological questionnaire were provided with the opportunity to fill it out onsite. When requested by participants, research assistants present at the location assisted with completion of the questionnaire and answered any existing questions. After form collection/completion, nurses obtained blood samples from each participant for both storage and sampling, and these were later analysed at the Human Immunobiology and Pathogenesis Laboratory at the NOVA NMS Centre for the Study of Chronic Diseases (CEDOC). Following the blood draw, participants were informed that their serological test results would be provided in ~4 days.

Once the serological test results were known, participants with positive serology were contacted by phone and invited to answer a questionnaire containing questions related to COVID-19. These included the following: “Since February of 2020, have you had one of these symptoms—presence of cough, fever, sore throat, lack of smell, lack of taste, headaches, body aches, shortness of breath (yes/no)? If yes, indicate the date of first symptoms. Since February of 2020, have you had contact with someone diagnosed or suspected of having COVID-19 (yes/no)? If yes, indicate the date. Since February, have you been travelling abroad (yes/no)? If yes, indicate the date of travel and country.”

This study protocol was submitted and approved by the NOVA NMS Ethics Committee, with all experiments and laboratory analysis following the provisions of the Declaration of Helsinki and the Good Clinical Practise guidelines of the International Conference on Harmonisation.

### Measures

The self-administered epidemiological questionnaire was designed to assess the presence of COVID-19 symptoms, the presence of chronic non-communicable diseases, the presence of depression and anxiety symptoms, and lifestyle habits. Specifically, the questionnaire was composed of questions regarding sociodemographic information (e.g., age, sex, school, level of education, job/position); anthropometric data (e.g., self-reported weight and height); COVID-19 symptoms in the previous 14 days (e.g., “Have you had one of these symptoms in the last 14 days: presence of cough, fever, sore throat, lack of smell, lack of taste, headaches, body aches, shortness of breath (yes/no)?”); presence of chronic non-communicable diseases (e.g., “Did any doctor tell you that you suffered from any of the following chronic diseases: high cholesterol levels, high blood pressure, allergy, gastrointestinal disease, mental disease, cardiac disease, diabetes, pulmonary disease, hyperuricemia, neoplastic disease, neurologic disease, urinary, rheumatic disease (yes/no)?”); lifestyle characteristics, including sleep hours (e.g., “How many hours do you sleep a day (on average)?”), alcohol intake (e.g., daily/occasionally/never), smoking habits (e.g., daily/occasionally/in the past/never); and chronic medication (e.g., “What medication do you take every day?”).

To assess the presence of anxiety and depression symptoms, we used the Hospital Anxiety and Depression Scale (HADS) (16), which has been validated for the Portuguese population (17). This instrument is composed of 14 questions that are rated on a four-point scale: 0 (not present) to 3 (significant symptoms). Anxiety and depression are assessed as separate components with seven items each. The questionnaire can be easily self-administered in ~2–5 min, showing good performance for inpatient and outpatient scenarios, as well as against other scales and clinical populations (18, 19). A score for each component was built by summation of the appropriate items, based on the participants' answers. Participants with scores ≥11 were classified as having anxiety and depression symptoms, which indicates “probable presence of the mood disorder,” as defined by Snaith (20). It is important to note, however, that are goal was not to diagnose depression or anxiety. Rather, our main aim was to assess the presence of depression and anxiety symptoms that could be present in the specific context of adaptation to the pandemic.

### Laboratory Procedures for Serological Testing

Blood samples were carefully transported and stored at the NOVA Biobank, where the serum was collected by centrifugation at 1,000 *x* g for 10 min. Plasma samples were aliquoted and stored in a Biobank freezer at −80°C. For subsequent analysis, the samples were thermally inactivated at 56°C for 60 min (21).

Serological tests were performed as described previously (21), within the scope of the Serology4COVID Consortium, which brings together the NMS CEDOC, the ITQB, and the IBET at NOVA, as well as the Gulbenkian Institute of Science (IGC), and the Institute of Molecular Medicine (IMM) (22). Using a modified enzyme-linked immunosorbent assay (ELISA) (21) that is based on a published protocol (23), the seropositivity cut-off point for IgG antibodies was set at 0.3987, with receiver operating characteristic (ROC) curve analysis determining a sensitivity of 94.74% [CI_95%_ (73.97, 99.87)] and a specificity of 99.53% [CI_95%_ (97.41, 99.99)] (21).

### Statistical Analysis

A descriptive analysis was performed, the results of which are presented as absolute frequencies, and prevalence estimates for IgG antibodies were computed as proportions. Participants with positive *vs*. negative serology results were compared, and prevalence estimates were computed for each predictor, including sex, age group, academic degree, faculty, body mass index (BMI), occupational group, smoking, alcohol consumption, and presence of chronic non-communicable comorbidities. The estimated prevalence of anxiety or depression symptoms was measured and compared between the two groups, and this was cross tabulated with sex, age group, academic degree, faculty, BMI, occupational group, smoking, alcohol consumption, and chronic non-communicable comorbidities.

Logistic regression models were fit to evaluate the association between presence of anxiety or depression symptoms and possible determinants. We also performed univariate analysis to test the following independent variables: demographic (e.g., sex, age group, school), lifestyles (e.g., tobacco, alcohol consumption, sleep hours), BMI, and comorbidities. The resulting variables with *p* < 0.2 were included in the multivariate models to assess the differences between individuals with and without depression or anxiety symptoms. The forward selection method was used to construct the multivariate logistic models, and possible confounding variables, such as sex and age group, were forced to remain in the model.

Significance was set at *p* < 0.05. Descriptive analysis, calculation of summary statistics, and statistical tests (Fisher, Chi-Squared) were performed using STATA IC v.16.1 (24). Regression models and graphics were generated using R Software (25).

## Results

A total of 1,627 subjects with a mean age of 42.0 ± 12.3 years were recruited, of which 1,624 were tested for the presence of IgG antibodies to SARS-CoV-2 (Figure 1). Response rate to the invitation email was of 49.2% (*n* = 1,627/3,308). Sample distribution is close to that of the study population with only minor variations in some categories likely due to differences from 2019 to 2020 (Table 1). Response rate to the questionnaire was over 99%, with only eight participants refusing to answer the questionnaire. Sample sizes for each variable response are presented, varying slightly due to non-responded questions. Logistic regression models were build using complete cases for all variables.

### Seroprevalence Against SARS-CoV-2 Among Employees of NOVA University

The estimated prevalence of IgG antibodies to SARS-CoV-2 among NOVA employees was found to be 3.1% (*n* = 50) (Table 2). When stratifying positive cases per school, we found that the NMS had the highest seroprevalence of IgG antibodies (6.2%), compared to the other schools. Conversely, a much lower seroprevalence was detected at the RUNL (0.9%), which had only one case (Table 3).

**Table 2:** Sociodemographic and health-related characterisation of NOVA University subjects, stratified based on SARS-CoV-2 serology results.

	**Total (*n* = 1,624)**	**Negative (*n* = 1,574)**	**Positive (*n* = 50)**
**Sex**	*n* = 1,624	*n* = 1,574	*n* = 50
Female	1,072 (66.0%)	1,038 (65.9%)	34 (68.0%)
Male	552 (34.0%)	536 (34.1%)	16 (32.0%)
**Age Group**	*n* = 1,616	*n* = 1,566	*n* = 50
<30	296 (18.3%)	285 (18.2%)	11 (22.0%)
(30, 39)	438 (27.1%)	423 (27.0%)	15 (30.0%)
(40, 49)	413 (25.6%)	401 (25.6%)	12 (24.0%)
(50, 59)	309 (19.1%)	300 (19.2%)	9 (18.0%)
≥60	160 (9.9%)	157 (10.0%)	3 (6.0%)
**Academic degree**	*n* = 1,557	*n* = 1,511	*n* = 46
PhD	633 (40.7%)	614 (40.6%)	19 (41.3%)
MSc	380 (24.4%)	368 (24.4%)	12 (26.1%)
BD	343 (22.0%)	333 (22.0%)	10 (21.7%)
Secondary School	131 (8.4%)	130 (8.6%)	1 (2.2%)
High School	43 (2.8%)	39 (2.6%)	4 (8.7%)
Complete Primary school	25 (1.6%)	25 (1.6%)	–
Primary School	2 (0.1%)	2 (0.1%)	–
**Faculty/Institute**	*n* = 1,598	*n* = 1,551	*n* = 47
ENSP	40 (2.6%)	40 (2.6%)	–
NMS	240 (15.0%)	225 (14.5%)	15 (31.9%)
FCSH	197 (12.3%)	193 (12.4%)	4 (8.5%)
FCT	447 (28.0%)	438 (28.2%)	9 (19.2%)
FD	31 (1.9%)	30 (1.9%)	1 (2.1%)
IHMT	46 (2.9%)	44 (2.8%)	2 (4.3%)
ITQB/IBET	289 (18.1%)	280 (18.1%)	9 (19.1%)
RUNL	113 (7.1%)	112 (7.2%)	1 (2.1%)
SBE	195 (12.2%)	189 (12.2%)	6 (12.8%)
**Body Mass Index (kg/m**^**2**^**)**	*n* = 1,583	*n* = 1,535	*n* = 48
Underweight (<18.5)	44 (2.8%)	42 (2.7%)	2 (4.2%)
Normal (18.5–24.9)	910 (57.5%)	881 (57.4%)	29 (60.4%)
Overweight (25–29.9)	475 (10.0%)	460 (30.0%)	15 (31.3%)
Obese (≥30)	154 (9.7%)	152 (9.9%)	2 (4.2%)
**Occupational group**	*n* = 1,559	*n* = 1,514	*n* = 45
Armed Forces	1 (0.1%)	1 (0.1%)	–
Executives/Directors/Managers	42 (2.7%)	42 (2.8%)	–
Scientific and Intellectual Areas	1,272 (81.6%)	1,235 (81.6%)	37 (82.2%)
Middle level	73 (4.7%)	69 (4.6%)	4 (8.9%)
Administrative	98 (6.3%)	97 (6.4%)	1 (2.2%)
Personal Services	20 (1.3%)	20 (1.3%)	–
Agriculture/Fishing	–	–	–
Industry/Construction	8 (0.5%)	8 (0.5%)	–
Machine operators	–	–	–
Non-qualified workers	45 (2.9%)	42 (2.8%)	3 (6.7%)
**Smoking**	*n* = 1,608	*n* = 1,558	*n* = 50
Never	907 (56.4%)	882 (56.6%)	25 (50.0%)
Daily	214 (13.3%)	210 (13.5%)	4 (8.0%)
Occasionally	101 (6.3%)	95 (6.1%)	6 (12.0%)
In the past	380 (23.6%)	365 (23.4%)	15 (30.0%)
DK/DA	6 (0.4%)	6 (0.4%)	–
**Alcohol**	*n* = 1,592	*n* = 1,543	*n* = 49
Never	198 (12.4%)	192 (12.4%)	6 (12.2%)
Daily	130 (8.2%)	124 (8.0%)	6 (12.2%)
Occasionally	1,255 (78.8%)	1,218 (78.9%)	37 (75.5%)
DK/DA	9 (0.6%)	9 (0.6%)	–
**Comorbidities**	*n* = 1,624	*n* = 1,574	*n* = 50
No	590 (36.3%)	568 (36.1%)	22 (44.0%)
Yes	1,034 (63.7%)	1,006 (63.9%)	28 (56.0%)
Presence of Chronic Diseases^*^			
Hypertension	190 (11.8%)	187 (11.9%)	3 (6.0%)
Diabetes	32 (2.0%)	31 (2.0%)	1 (2.0%)
Cholesterol	266 (16.6%)	258 (16.6%)	8 (16.0%)
Pulmonary	58 (3.6%)	57 (3.7%)	1 (2.0%)
Cardiac	101 (6.3%)	99 (6.3%)	2 (4.0%)
Thrombose	8 (0.5%)	8 (0.5%)	–
Digestive	246 (15.3%)	238 (15.3%)	8 (16.0%)
Neurological	180 (11.2%)	175 (11.2%)	5 (10.2%)
Allergies	571 (35.5%)	556 (35.7%)	15 (30.0%)
Oncological	44 (2.8%)	43 (2.8%)	1 (2.0%)
Hyperuricemia	25 (1.6%)	23 (1.5%)	2 (4.0%)
Urinary	74 (4.8%)	74 (4.8%)	–
Rheumatic	82 (5.3%)	80 (5.4%)	2 (4.1%)
**Chronic medication**	*n* = 1,616	*n* = 1,566	*n* = 50
No	975 (60.3%)	940 (60.0%)	35 (70.0%)
Yes	641 (39.7%)	626 (40.0%)	15 (30.0%)
**Presence of Anxiety**	121 (8.1%)	114 (7.8%)	7 (16.3%)
**Presence of Depression**	32 (2.1%)	30 (2.1%)	2 (4.6%)

* *Sample size is not constant due to missing values in: Hypertension (n = 1,611), Diabetes (n = 1,606), Cholesterol (n = 1,605), Pulmonary (n = 1,612), Cardiac (n = 1,612), Thrombose (n = 1,611), Digestive (n = 1,606), Neurological (n = 1,608), Allergies (n = 1,609), Oncological (n = 1,575), Hyperuricemia (n = 1,585), Urinary (n = 1,583), Rheumatic (n = 1,545), Presence of Anxiety (n = 1,500), Presence of Depression (n = 1,507)*.

*Negative SARS-CoV-2 serology: Hypertension (n = 1,561), Diabetes (n = 1,556), Cholesterol (n = 1,555), Pulmonary (n = 1,562), Cardiac (n = 1,562), Thrombose (n = 1,561), Digestive (n = 1,556), Neurological (n = 1,559), Allergies (n = 1,559), Oncological (n = 1,525), Hyperuricemia (n = 1,535), Urinary (n = 1,533), Rheumatic (n = 1,496), Presence of Anxiety (n = 1,457), Presence of Depression (n = 1,463)*.

*Positive SARS-CoV-2 serology: Neurological (n = 49), Rheumatic (n = 49), Presence of Anxiety (n = 43), Presence of Depression (n = 44)*.

*DK/DA, Didn't know/ Didn't answer*.

**Table 3:** Seroprevalence University sample distribution by organic unit.

	**NOVA 2019**	**Participation rate (%)**	**Seroprevalence (%)**
**Total**	***n*** **= 3,308**	***n*** **= 1,624 (49.2%)**	***n*** **= 50 (3.1%)**
**Faculty/Institute**		*n* = 1,598	*n* = 47
National Public Health School	67	40 (59.7%)	–
Medical School	801	240 (30.0%)	15 (6.2%)
Social and Human Sciences School	620	197 (31.8%)	4 (2.0%)
Sciences and Technology School	753	447 (59.4%)	9 (2.0%)
Law School	69	31 (44.9%)	1 (3.2%)
Hygiene and Tropical Medicine Institute	109	46 (42.2%)	2 (4.3%)
Chemical and Biological Technology Institute^#^	195	289 (Not comparable)	9 (3.1%)
Rectorate	172	113 (65.7%)	1 (0.9%)
Business and Economics School	438	195 (44.5%)	6 (3.1%)
Information and Management School	84	–	–

# *Chemical and Biological Technology Institute (n = 225) + Experimental and technological Biology Institute (IBET) (n = 67)*.

Those with positive serology had similar characteristics to participants with a negative result, being mostly women (68.0 *vs*. 65.9%, respectively) and with ages between 17 and 66 years (mean age of 39.3 ± 11.95 vs. 42.0 ± 12.28 years, respectively). Twenty participants (43.5%) with a positive result reported having at least one symptom suggestive of COVID-19, and 10.9% reported contact with diagnosed patients between February and June of 2020. In that same period, 12 participants with positive serology travelled abroad (26.1%), with Spain and England being the most frequent destinations.

### Mental Health and Comorbidities of NOVA University Employees During the COVID-19 Pandemic

A total of 63.7% of participants reported having at least one chronic non-communicable disease, with 39.7% taking medication for at least one chronic condition (Table 2). Allergies were the most common chronic condition (35.5%), followed by high cholesterol levels (16.6%).

**Figure 2:**
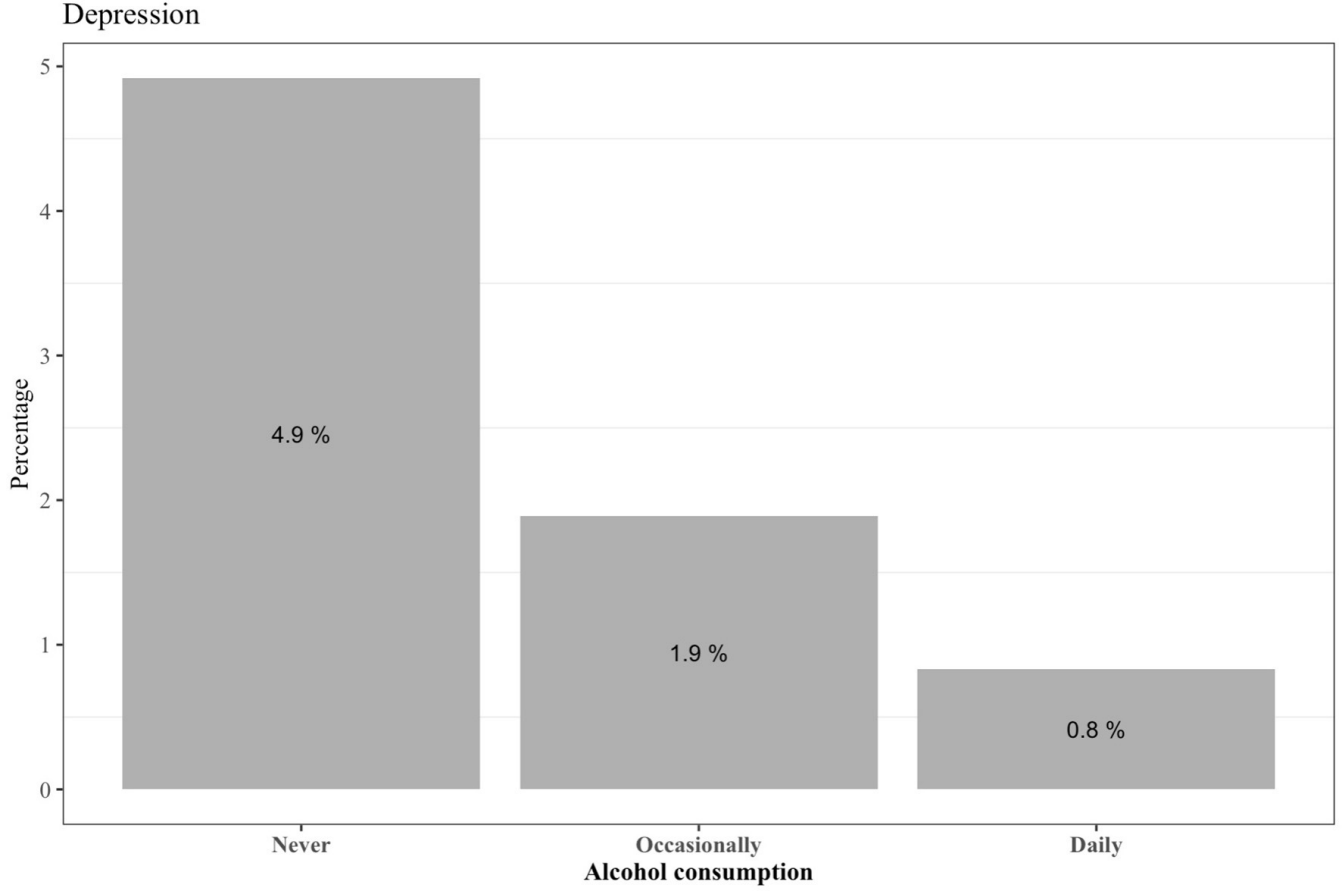
Prevalence of depression symptoms per alcohol consumption levels (*n* = 32).

The overall prevalence of depression symptoms was 2.1%, while that of anxiety symptoms was 8.1%, both significantly affecting women more than men (depression: 84.4 vs. 15.6%, respectively, Fisher's exact *p* = 0.024; anxiety: 87.6 vs. 12.4%, respectively, Fisher's exact *p* = 0.000) (Table 4). Alcohol consumption was also negatively associated with the presence of depression symptomatology (Pearson Chi^2^ = 8.267, *p* = 0.041), with those reporting occasional and daily consumption levels having lower depression frequencies relative to participants who did not drink (Figure 2).

**Table 4:** Sociodemographic and health-related characterisation of NOVA University subjects, stratified based on mental health assessment results.

	**Total (*n* = 1,627)**	**Presence of depression (*n* = 32)**	**Presence of anxiety (*n* = 121)**
**Sex**	*n* = 1,627	*n* = 32	*n* = 121
Female	1,074 (66.0%)	27 (84.4%)	106 (87.6%)
Male	553 (34.0%)	5 (15.6%)	15 (12.4%)
**Age Group**	*n* = 1,619	*n* = 32	*n* = 121
<30	297 (18.3%)	3 (9.4%)	28 (23.1%)
(30, 39)	438 (27.1%)	7 (21.9%)	30 (24.8%)
(40, 49)	414 (25.6%)	10 (31.3%)	36 (30.0%)
(50, 59)	310 (19.1%)	10 (31.3%)	20 (16.5%)
≥60	160 (9.9%)	2 (6.2%)	7 (5.8%)
**Academic degree**	*n* = 1,560	*n* = 31	*n* = 120
PhD	634 (40.6%)	13 (42.0%)	47 (39.2%)
MSc	381 (24.4%)	9 (29.0%)	29 (24.2%)
BD	344 (22.1%)	4 (12.9%)	30 (25.0%)
Secondary School	131 (8.4%)	5 (16.1%)	20 (8.3%)
High School	43 (2.7%)	–	4 (3.3%)
Complete primary school	25 (1.6%)	–	–
Primary School	2 (0.1%)	–	–
**Faculty/Institute**	*n* = 1,601	*n* = 32	*n* = 117
ENSP	40 (2.5%)	1 (3.1%)	3 (2.6%)
NMS	240 (15.0%)	–	8 (6.8%)
FCSH	197 (12.3%)	6 (18.8%)	19 (16.2%)
FCT	448 (27.9%)	13 (40.6%)	32 (27.4%)
FD	31 (1.9%)	–	7 (6.0%)
IHMT	46 (2.9%)	2 (6.2%)	3 (2.6%)
ITQB/IBET	290 (18.1%)	4 (12.5%)	25 (21.4%)
RUNL	114 (7.1%)	1 (3.1%)	9 (7.7%)
SBE	195 (12.2%)	5 (15.6%)	11 (9.4%)
**Body Mass Index (kg/m**^**2**^**)**	*n* = 1,586	*n* = 31	*n* = 118
Underweight (<18.5)	44 (2.8%)	1 (3.2%)	4 (3.4%)
Normal (18.5–24.9)	912 (57.5%)	16 (51.6%)	69 (58.5%)
Overweight (25–29.9)	475 (30.0%)	8 (25.8%)	28 (23.7%)
Obese (≥30)	155 (9.8%)	6 (19.4%)	17 (14.4%)
**Occupational group**	*n* = 1,562	*n* = 30	*n* = 118
Armed Forces	1 (0.1%)	–	–
Executives/Directors/Managers	42 (2.7%)	–	5 (4.2%)
Scientific and Intellectual Areas	1,275 (81.6%)	21 (70.0%)	84 (71.2%)
Middle level	73 (4.7%)	2 (6.7%)	5 (4.2%)
Administrative	98 (6.3%)	4 (13.3%)	7 (5.9%)
Personal Services	20 (1.3%)	–	1 (0.9%)
Agriculture/Fishing	–	–	–
Industry/Construction	8 (0.5%)	–	3 (2.5%)
Machine operators	–	–	–
Non-qualified workers	45 (2.9%)	3 (10.0%)	13 (11.0%)
**Smoking**	*n* = 1,611	*n* = 32	*n* = 120
Never	910 (56.5%)	14 (43.8%)	55 (45.8%)
Daily	214 (13.3%)	7 (21.9%)	21 (17.5%)
Occasionally	101 (6.3%)	3 (0.4%)	11 (9.2%)
In the past	380 (23.6%)	8 (25.0%)	32 (26.7%)
DK/DA	6 (0.4%)	–	1 (0.8%)
**Alcohol**	*n* = 1,595	*n* = 32	*n* = 121
Never	198 (12.4%)	9 (28.1%)	19 (15.7%)
Daily	131 (8.2%)	1 (3.1%)	8 (6.6%)
Occasionally	1,257 (78.8%)	22 (68.8%)	94 (77.7%)
DK/DA	9 (0.6%)	–	–
**Comorbidities**	*n* = 1,627	*n* = 32	*n* = 121
No	591 (36.3%)	4 (12.5%)	25 (20.7%)
Yes	1,036 (63.7%)	28 (87.5%)	96 (79.3%)
Presence of Chronic Diseases^*^			
Hypertension	191 (11.8%)	5 (15.6%)	15 (12.4%)
Diabetes	32 (2.0%)	–	4 (3.3%)
Cholesterol	267 (16.6%)	9 (28.1%)	25 (20.7%)
Pulmonary	58 (3.6%)	1 (3.1%)	3 (2.5%)
Cardiac	101 (6.3%)	9 (28.1%)	16 (12.2%)
Thrombose	8 (0.5%)	1 (3.2%)	–
Digestive	247 (15.4%)	10 (31.3%)	31 (25.8%)
Neurological	180 (11.2%)	7 (21.9%)	29 (24.0%)
Allergies	572 (35.5%)	16 (50.0%)	59 (48.8%)
Oncological	44 (2.8%)	3 (9.7%)	4 (3.4%)
Hyperuricemia	25 (1.6%)	2 (6.3%)	4 (3.3%)
Urinary	74 (4.7%)	3 (9.4%)	6 (5.0%)
Rheumatic	82 (5.3%)	5 (16.7%)	13 (11.3%)

* *Sample size is not constant due to missing values in: Hypertension (n = 1,614), Diabetes (n = 1,609), Cholesterol (n = 1,608), Pulmonary (n = 1,615), Cardiac (n = 1,615), Thrombose (n = 1,614), Digestive (n = 1,609), Neurological (n = 1,611), Allergies (n = 1,612), Oncological (n = 1,578), Hyperuricemia (n = 1,588), Urinary (n = 1,586), Rheumatic (n = 1,548)*.

*Participants with depression symptoms: Diabetes (n = 31), Thrombose (n = 31), Oncological (n = 31), Rheumatic (n = 30)*.

*Participants with anxiety symptoms: Diabetes (n = 120), Thrombose (n = 120), Digestive (n = 120), Oncological (n = 119), Hyperuricemia (n = 120), Urinary (n = 120), Rheumatic (n = 115)*.

*DK/DA, Didn't know/ Didn't answer*.

**Figure 3:**
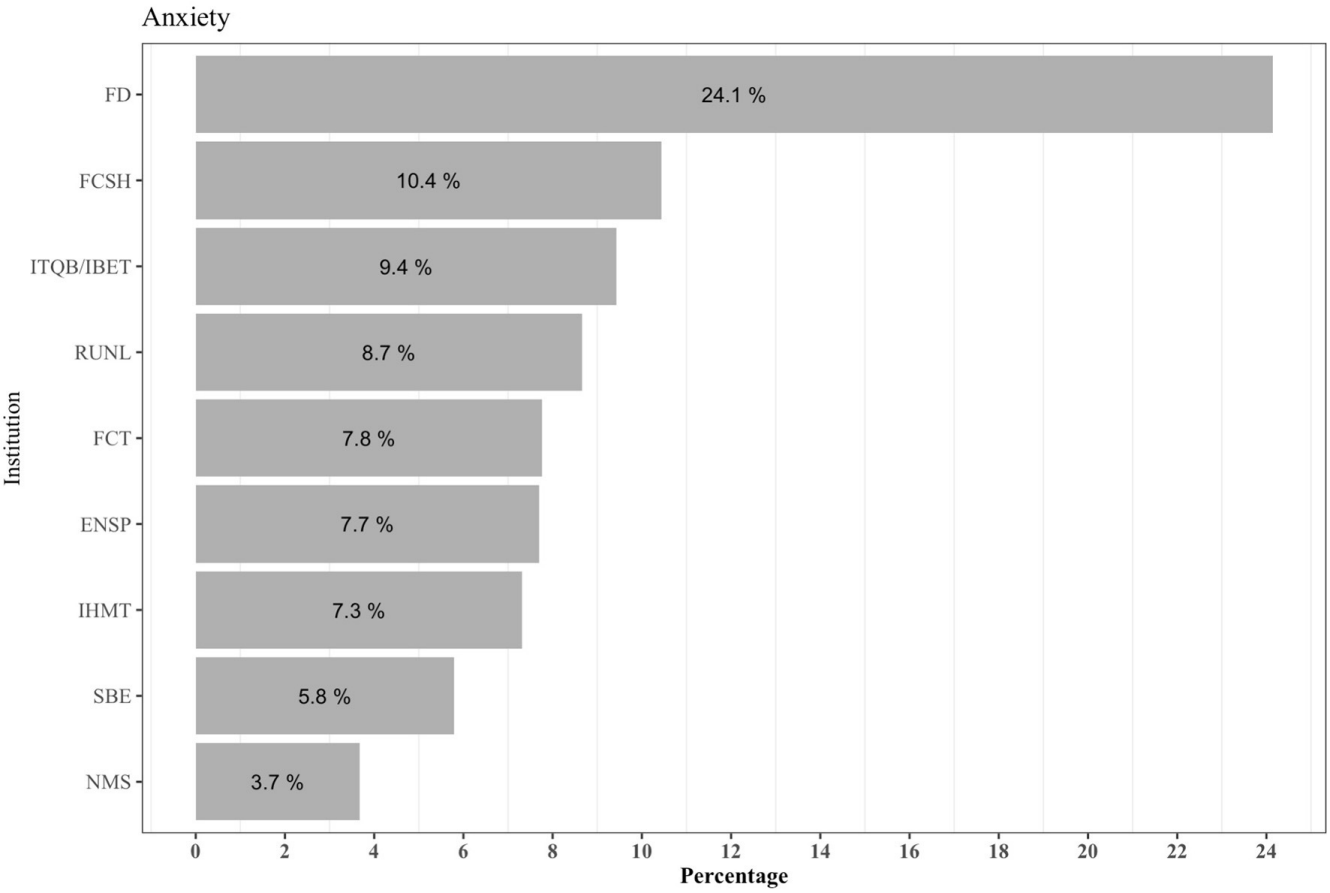
Prevalence of anxiety symptoms based on the number of individuals per organic unit (*n* = 117).

NOVA's Law School (FD) had the highest prevalence of anxiety symptoms (24.1%) (Figure 3), with all groups differing significantly (Pearson Chi^2^ = 19.605, *p* = 0.012; Table 4). Further, although the differences were not statistically significant, we noted a higher prevalence of depression (4.6%) and anxiety (16.3%) among participants with positive serology, relative to those who were negative (2.1 and 7.8%, respectively).

Logistic regression analysis to identify factors associated with depression symptoms revealed statistically significant negative associations between depression symptoms and an increase in daily sleep hours (OR = 0.464, *p* < 0.001) and between depression symptoms and occasional alcohol drinkers in comparison to non-drinkers (OR = 0.405, *p* = 0.045) (Table 5). In particular, a mean increase in 1 h of sleep per day was found to decrease the odds of depression symptomatology by 53.6% (1–0.464). The odds of depression symptoms decreased by 59.5% for occasional alcohol drinkers compared to those who never drink alcohol. The odds of depression symptoms were 2.7 times higher for those with comorbidities, relative to those without comorbidities. We further note that although sex and age groups were not significantly associated with depression when adjusting for the remaining predictors, we considered them important and kept them in the model.

**Table 5:** Logistic regression analysis to identify factors associated with the presence of depression symptoms in NOVA employees.

**Depression**	**β**	**OR**	**95% CI**	***z***	***p***
**Sex**					
Female (ref)	–	–	–	–	–
Male	−0.753	0.471	(0.154, 1.194)	−1.469	0.142
**Age group**					
<30 (ref)	–	–	–	–	–
(30, 39)	0.256	1.292	(0.332, 6.236)	0.356	0.722
(40, 49)	0.633	1.883	(0.553, 8.603)	0.937	0.349
(50, 59)	0.615	1.850	(0.503, 8.767)	0.875	0.382
≥60	−1.116	0.328	(0.015, 2.617)	−0.941	0.347
**Sleep hours**	−0.768	0.464	(0.311, 0.691)	−3.792	<0.001*
**Alcohol**					
Never (ref)	–	–	–	–	–
Daily	−1.448	0.235	(0.012, 1.414)	−1.320	0.187
Occasionally	−0.905	0.405	(0.172, 1.034)	−2.001	0.045*
**Comorbidities**	1.009	2.743	(1.018, 9.554)	1.814	0.070
(*n* = 1,345)				AIC: 259.29

*OR, Odds Ratio; β, Regression coefficients; 95% CI, 95% Confidence intervals; z, z-test statistics; p, p-values; AIC, Akaike Information Criteria*.

*Regression coefficients, odds ratios (ORs), 95% confidence intervals (CIs), z-test values, and Wald test p-values of the model (symptoms suggestive of depression vs. no symptoms suggestive of depression) are shown*.

The presence of anxiety symptoms showed a significant negative association with being male (OR = 0.246, *p* < 0.001), being in older age groups compared to the <30 years-old group, sleeping more hours (OR = 0.452, *p* < 0.001), and belonging to NMS (OR = 0.410, *p* = 0.04) vs. the FCT. Conversely, the presence of comorbidities (OR = 2.888, *p* < 0.001) and belonging to the FD (OR = 6.275, *p* < 0.001) vs. the FCT (Table 6) were positively associated with anxiety symptoms.

**Table 6:** Logistic regression analysis to identify factors associated with the presence of anxiety symptoms in NOVA employees.

**Anxiety**	**β**	**OR**	**95% CI**	***z***	***p***
**Sex**					
Female (ref)	–	–	–	–	–
Male	−1.402	0.246	(0.129, 0.438)	−4.519	<0.001*
**Age group**					
<30 (ref)	–	–	–	–	–
(30, 39)	−0.673	0.510	(0.274, 0.949)	−2.134	0.032*
(40, 49)	−0.640	0.527	(0.281, 0.990)	−1.999	0.046*
(50, 59)	−1.092	0.336	(0.158, 0.691)	−2.921	0.003*
≥60	−2.332	0.097	(0.022, 0.308)	−3.555	<0.001*
**Sleep hours**	−0.795	0.452	(0.353, 0.573)	−6.467	<0.001*
**Faculty/Institution**					
FCT (ref)	–	–	–	–	–
ENSP	−0.105	0.900	(0.138, 3.365)	−0.135	0.892
NMS	−0.891	0.410	(0.165, 0.922)	−2.053	0.040*
FCSH	0.353	1.423	(0.707, 2.796)	1.012	0.312
FD	1.837	6.275	(2.104, 17.473)	3.439	<0.001*
IHMT	−0.537	0.584	(0.087, 2.287)	−0.674	0.500
ITQB/IBET	0.139	1.150	(0.610, 2.153)	0.435	0.663
RUNL	0.039	1.040	(0.413, 2.398)	0.089	0.929
SBE	−0.411	0.663	(0.277, 1.458)	−0.982	0.326
**Comorbidities**	1.061	2.888	(1.720, 5.084)	3.854	<0.001*
(*n* = 1,332)				AIC: 645.88

*OR, Odds Ratio; β, Regression coefficients; 95% CI, 95% Confidence intervals; z, z-test statistics; p, p-values; AIC, Akaike Information Criteria*.

*Regression coefficients, odds ratios (ORs), 95% confidence intervals (CIs), z-test values, and Wald test p-values of the model (symptoms suggestive of anxiety vs. no symptoms suggestive of anxiety) are shown*.

## Discussion

The low prevalence of SARS-CoV-2 antibodies detected in this study (3.1%) is consistent with other reports (26, 27). Our results also align with the 2.9% estimated prevalence reported from a serological study of the Portuguese population performed in July of 2020 (28), indicating that NOVA employees were not at a higher risk of contracting the disease. However, our prevalence rate is somewhat higher than that detected by the COVID-19 National Serological Panel (1.9%) in September 2020 (29). The difference found in the prevalence of SARS-CoV-2 antibodies between our study's and the COVID-19 National Serological Panel one could potentially be explained by the subsequent: government-enforced national lockdown, social distancing, and the fast transition to distance learning and telework. Nevertheless, it is worth noting that the low prevalence found in this study is also indicative that, at the time of testing, there was no active community transmission of COVID-19 within NOVA University.

Among the surveyed schools, the Medical School (NMS) had the highest seroprevalence of IgG anti-SARS-CoV-2 antibodies (6.2%), followed by the Tropical Medicine Institute (IHMT; 4.3%). This result might be due to the fact that workers in these schools were practising clinicians, who were not confined and, thus, had a higher risk of being exposed to the virus. The lower seroprevalence detected in other schools, with a maximum of 3.2%, is more in line with national figures (28). Similarly, a serological study at the University of Athens reported higher seroprevalence in health science schools during June/July of 2020, although this was not statistically significant (30). These findings suggest that the massive reduction of in-person academic activities effectively prevented the spread of SARS-CoV-2, leading us to propose additional measures to further minimise inter-person contacts. Our study informed the NOVA University management structure of the need to implement a stricter contingency plan in the NMS, while other schools within the university continued to follow Government mitigation guidelines.

In the United States, reopening of campuses was considered safe and to have minimal impact on community spread if positivity rates were under 5%, provided regular testing was implemented to monitor infection rates (31). Universities could reopen with regular testing and measures to prevent physical contact. Such measures include arranging circuits to ensure physical distancing and limiting campus class attendance by employing blended classes (i.e., online plus in-person), combined with standard good practises, such as mandatory mask usage, regular disinfection of hands and surfaces, and temperature measurements Other recommended measures include publishing daily or weekly metrics regarding safety protocols and the number of cases and tests at the university, independent from regional data, to understand differences between academic and local communities (32). Such communication would also provide valuable feedback to university personnel regarding the impact of safety measures, which is expected to favour adherence to contingency initiatives (33).

Our findings showed a positivity rate of 3.1% which allowed us to recommend at NOVA University's Governing Body a safe campus reopening. Even though in our university most infections took place outside the campus, a rigorous contingency plan was developed to lower the risk of COVID-19 spread on campus. We have participated in the development of NOVA COVID-19 Contingency Plan and proposed measures such as the progressive return to in-person work, massive testing of all university employees, enforcing indoor room occupancy limits; defining circuits of entry, occupation, exit and circulation; and implementing mandatory face mask usage when at NMS.

Based on our results, it was possible to define strategies that would need to be developed, implemented, and adjusted to prepare for the upcoming academic year (2020–2021) and effectively prevent the spread of COVID-19 infection.

Our mental health assessment of the NOVA academic community further revealed a relatively low prevalence of anxiety and depression symptoms when compared with other studies of the general population (34–36), and even in universities before the pandemic (37, 38). Nevertheless, these rates were not negligible, and we report a number of key findings. The results revealed a high prevalence of moderate-to-severe anxiety symptoms in the institution's community. Following this evidence, the Rectory was duly informed of the scenery found by the present study. Accordingly, our team of researchers launched an alert on the need to respond to the disturbing mental health situation, having suggested creating a crisis office service. This crisis office service would react as the first line to the urgent needs of mental health, in a logic of psychological first aid, rooted in a model of seven steps: T – triage; P – Protect; S – Stabilise; I – Inform; E – Educate; C – Connect; HO - Help Organise, and R – Refer (39, 40). In addition, it was also suggested the creation of a mental health intervention office or decentralised offices, in a structural and long-lasting service logic, including public health and mental health professionals, to promote global occupational health.

Although we found no significant differences between prevalence of depression and anxiety in seropositive *vs*. negative individuals, the presence of these symptoms may reflect a general concern and fear related to the pandemic and pandemic-associated constraints (41). However, further research is needed to address the overall lack of knowledge relating to the impact that this pandemic has had on mental health of university personnel and to identify its effects on their well-being and life satisfaction. In addition to pandemic-induced worry about family, friends, and infected acquaintances, as well as uncertainty and fear for the future, the COVID-19-related lockdowns have created social isolation, which is an established risk factor for psychological harm (42, 43). In their systematic review based on 25 studies, Prati et al. found a small, yet significant, effect of the COVID-19 lockdowns on mental health symptoms within the general population, namely depression and anxiety, although the authors cautiously note the high heterogeneity of effects (44). Moreover, anxiety and depression have been reported to be more frequent in women than in men due to biological differences, as well as the lack of social attitudes, policies, and support systems to promote equality (45). Among the participants that showed anxiety symptoms, the majority were from the Law School (FD). We believe that this may be due to the organisational culture, management, or leadership differences, although we also note that this school had the smallest sample size (*n* = 29), thus showing greater variation.

Based on our logistic regression analysis, alcohol consumption was significantly associated with lower risk of depression, male gender and increased age were significantly associated with decreased anxiety, and sleep hours were significantly associated with lower risk of both depression and anxiety symptoms. In contrast, the presence of comorbidities was positively associated with anxiety. Other studies have described a J-shape association between alcohol consumption and depression symptoms, i.e., a lower risk of depression for light and moderate drinkers when compared to extreme drinkers or non-drinkers (46, 47), even when adjusted for past drinking (48). This negative association with depression for occasional drinkers may be explained by unmeasured social behaviour. That is, non-drinkers might be reluctant to attend social gatherings that involve alcohol use, and if limited social opportunities are, in fact, due to the decision not to drink, this can lead to increased depression and social anxiety (48). However, it is unclear whether this was a factor here, given the increased distancing and general decrease in social activities for both drinkers and non-drinkers during the pandemic. Our results also align with some previously reported risk factors for depression and anxiety symptoms, such as female gender (49, 50) and comorbidities (51). Workload has also been reported to be positively associated with the presence of psychological disturbances, including anxiety and depression (52, 53). Moreover, although anxiety and depression symptoms were not present in any of the medical staff in our sample, the majority of participants worked in scientific and intellectual areas, limiting statistical power to infer occupational group differences. Accordingly, our results highlight the need for preventive intervention to support academic communities and promote mental health, such as by creating mobile support lines targeting the academic community (54). Overall, a major strength of this study is that, unlike the national serological study, we have obtained serological information to measure SARS-CoV-2 exposure in an academic environment during the first COVID-19 wave, and in conjunction, we further provide a robust characterisation of the general mental health of the study population. Study limitations include the fact that our results were based on self-reports of diseases and COVID-19 symptoms, and therefore, we cannot ensure that participants paid sufficient attention to these questions, and thus the veracity of their answers, due to daily life stresses and time constraints.

## Conclusion

This study aimed to estimate the seroprevalence of SARS-CoV-2 antibodies and provide insight on the mental health status of workers in an academic environment. Our findings contribute to the general knowledge on prevalence of SARS-CoV-2 antibodies, which we found to be close to the national estimated prevalence for July (3.1 vs. 2.9%, respectively), indicating that NOVA employees were not at higher risk of having COVID-19.

The results of this study allowed us to recommend at NOVA University's Governing Body a safe campus reopening and prepare campus contingency policies to deal with the pandemic and to implement reopening plans. Although we found a lower prevalence of anxiety and depression symptoms than in other general population studies and even academic studies before the pandemic, these symptoms are frequent in academic environments and are consistently higher in certain groups (e.g., women), suggesting that the development of strategies to prevent mental illness and provide increased support to at-risk members of the academic community should be a priority. NOVA rectory was duly informed of the scenery found by the present study. Accordingly, our team of researchers launched an alert on the need to respond to the disturbing mental health situation, having suggested creating a crisis office service.

## Data Availability Statement

The raw data supporting the conclusions of this article will be made available by the authors, without undue reservation.

## Ethics Statement

The studies involving human participants were reviewed and approved by ethical committee of NMS|FCM (CEFCM). Written informed consent to participate in this study was provided by the participants' legal guardian/next of kin.

## Author Contributions

AR and HC: designed the epidemiological survey. JG and HS: customised the ELISA assay. DL, AH, CN-d-S, JE, SA, SB, RdS, AR, and HC: performed the epidemiological survey. RdS, HS, HC, AR, and JB: conceived the project and supervised the study. DL: analysed the data. DL and AH: interpreted the data and wrote the manuscript. CN-d-S, MS-D, HC, AR, and HS: revised the manuscript. All authors contributed to the article and approved the submitted version.

## Conflict of Interest

The authors declare that the research was conducted in the absence of any commercial or financial relationships that could be construed as a potential conflict of interest.
