# Supplementary material for: Staff SARS-CoV-2 Seroprevalence and Mental Health as Key Factors in University Response to COVID-19 Pandemic
Source: Front Public Health. 2021 Jun 16;9:689919. doi: 10.3389/fpubh.2021.689919 (PMC8241921; doi:10.3389/fpubh.2021.689919)
Supplement: Supplementary file 1 [file Table_1.pdf]

Supplementary Material

Supplementary Table 1: Participant's affiliation with NOVA University.

ENSP	National Public Health School
NMS	Medical School
FCSH	Social and Human Sciences School
FCT	Sciences and Technology School
FD	Law School
IHMT	Hygiene and Tropical Medicine Institute
ITQB	Chemical and Biological Technology Institute
IBET	Experimental and Technological Biology Institute
RUNL	Rectorate
SBE	Business and Economics School

Supplementary Table 2: Number of days and per location.

	NMS	FCT	IHMT	ITQB/IBET	RUNL	SBE
Nº of days	1	3	1	2	4	1

Supplementary Table 3: Number of people tested per day.

June	15th	16th	17th	18th	19th	22nd	23rd	24th	25th	26th	29th	30th
N	9	130	129	230	155	162	68	122	200	122	141	156
